# What is the impact of hospital and surgeon volumes on outcomes in rectal cancer surgery?

**DOI:** 10.1111/codi.16745

**Published:** 2023-09-13

**Authors:** Jemma M. Boyle, Jan van der Meulen, Angela Kuryba, Thomas E. Cowling, Michael S. Braun, Ajay Aggarwal, Kate Walker, Nicola S. Fearnhead

**Affiliations:** ^1^ Department of Health Services Research and Policy London School of Hygiene and Tropical Medicine London UK; ^2^ Clinical Effectiveness Unit Royal College of Surgeons of England London UK; ^3^ Department of Oncology The Christie NHS Foundation Trust Manchester UK; ^4^ School of Medical Sciences University of Manchester Manchester UK; ^5^ Department of Oncology Guy's and St. Thomas' NHS Foundation Trust London UK; ^6^ Department of Colorectal Surgery Cambridge University Hospitals Cambridge UK

**Keywords:** centralisation, rectal cancer, specialization, volume‐outcome

## Abstract

**Aim:**

Evidence for a positive volume–outcome relationship for rectal cancer surgery is unclear. This study aims to evaluate the volume–outcome relationship for rectal cancer surgery at hospital and surgeon level in the English National Health Service (NHS).

**Method:**

All patients undergoing a rectal cancer resection in the English NHS between 2015 and 2019 were included. Multilevel multivariable logistic regression was used to model relationships between outcomes and mean annual hospital and surgeon volumes (using a linear plus a quadratic term for volume) with adjustment for patient characteristics.

**Results:**

A total of 13 858 patients treated in 166 hospitals were included. Six hospitals (3.6%) performed fewer than 10 rectal cancer resections per year, and 381 surgeons (45.0%) performed fewer than five such resections per year. Patients treated by high‐volume surgeons had a reduced length of stay (*p* = 0.016). No statistically significant volume–outcome relationships were demonstrated for 90‐day mortality, 30‐day unplanned readmission, unplanned return to theatre, stoma at 18 months following anterior resection, positive circumferential resection margin and 2‐year all‐cause mortality at either hospital or surgeon level (*p* values > 0.05).

**Conclusion:**

Almost half of colorectal surgeons in England do not meet national guidelines for rectal cancer surgeons to perform a minimum of five major resections annually. However, our results suggest that centralizing rectal cancer surgery with the main focus of increasing operative volume may have limited impact on NHS surgical outcomes. Therefore, quality improvement initiatives should address a wider range of evidence‐based process measures, across the multidisciplinary care pathway, to enhance outcomes for patients with rectal cancer.

**What does this paper add to the literature?**

This study aims to overcome previous methodological limitations and provide current national evidence on the volume–outcome relationship (hospital and surgeon level) for rectal cancer surgery in the English NHS. No clear evidence for a volume–outcome relationship was demonstrated at hospital or surgeon level across a wide range of outcome measures.

## INTRODUCTION

An increasing body of evidence has shown improved postoperative and long‐term oncological outcomes for hospitals and surgeons performing higher volumes of more complex surgical procedures including oesophagectomy, gastrectomy, pancreatectomy and hepatectomy [1, 2, 3, 4]. As a result, many countries have adopted surgical specialization and provision of these surgical procedures in selected hospitals (a process sometimes referred to as ‘centralization’) in order to increase institutional case volumes [5, 6]. The provision of oesophago‐gastric cancer services in England via a ‘hub‐and‐spoke’ model coincided with a reduction in postoperative mortality from 7.4% to 2.5%, although this was not explained by increases in hospital volume alone [7].

Management of rectal cancer is becoming increasingly challenging due to the complexity of available treatment options and the need for multidisciplinary team (MDT) input to make decisions about neoadjuvant and adjuvant therapies, local excision, watch‐and‐wait strategies, offering organ preservation, surgical approach (including robotic access), surgical procedure (appropriateness of sphincter‐sparing surgery weighed against poor functional outcomes), and the use of temporary stomas. However, there is conflicting evidence for a volume–outcome relationship for rectal cancer at both hospital and surgeon level [8, 9, 10].

To date, there have been significant methodological limitations with studies evaluating the volume–outcome relationship for rectal cancer [11]. This includes grouping volumes into arbitrary categories, which leads to a reduction in the statistical power to detect a volume–outcome relationship. Categorizing of volumes also makes it more difficult to pool results because the volume thresholds used vary widely. The relevant outcomes, especially those important to patients, have not been evaluated adequately. There is significant heterogeneity in study populations (e.g. whether to include distal sigmoid colon cancer, patients with advanced stage disease, types of operations or emergency surgery) and patient characteristics included in risk‐adjustment models. In addition, retrospective analyses often use data from the 1990s and early 2000s, when laparoscopic surgery was not routinely used and outcomes, such as postoperative mortality, were generally worse [12, 13].

The lack of clear evidence on a volume–outcome relationship has resulted in variation in national guidelines on the recommendations for minimum annual volume of rectal cancer resections at an institutional level (e.g. 10 in England, 20 in Germany and the Netherlands, and 21 in the United States) and per surgeon (e.g. 5 in England and 10 in Germany) [14, 15].

This large national study aims to address this existing gap in evidence for the volume–outcome relationship. We used contemporary linked national clinical datasets including all hospitals providing rectal cancer surgery in the English National Health Service (NHS), with no exclusions, and case ascertainment beyond 95% of all diagnosed cases. Using these rich and complete data, we performed comprehensive risk adjustment and report an extensive panel of outcome measures, modelling volume as a continuous variable, and ensuring surgeon‐level information is robust through cross‐validation of information between data sources.

## METHOD

### Data sources

This study used data from the National Bowel Cancer Audit (NBOCA) [16], Hospital Episode Statistics (HES) [17], Radiotherapy Dataset (RTDS) [18], and Office for National Statistics (ONS) mortality data [19] linked at patient‐level for patients with a primary diagnosis of rectal cancer in the English NHS [International Classification of Diseases, 10th edition (ICD‐10) code C20].

#### National Bowel Cancer Audit

The NBOCA is a prospective mandatory database for all patients newly diagnosed with colorectal cancer in the English NHS. NBOCA case ascertainment is above 95% when compared with HES and National Cancer Registration and Analysis Service (NCRAS) ‘gold standard’ data [20]. Patients diagnosed within the private sector and undergoing major resection in an NHS hospital were included. Patients diagnosed and treated entirely in the private sector were not captured, but represent a small number of patients.

Data items from the NBOCA were used to determine sex, age, Eastern Cooperative Oncology Group (ECOG) performance status, pathological staging according to the TNM system, American Society of Anesthesiologists (ASA) score, date of surgery, surgical procedure, surgical urgency (elective/scheduled or emergency/urgent), and surgical access.

#### Hospital Episode Statistics

The HES dataset is a national administrative dataset of all admissions to English NHS hospitals [17]. HES provided information on the number of comorbidities according to the Royal College of Surgeons of England Comorbidity score [21], socioeconomic deprivation reported as quintiles of the national distribution of the Index of Multiple Deprivation (IMD) [22], and ethnicity.

#### Radiotherapy Dataset

Radiotherapy information was obtained from linkage to the RTDS and included whether the patient received radiotherapy and whether this was short‐ or long‐course based on prior methodology using the number of fractionations and time between radiotherapy and surgery [23]. The study timeframe pre‐dated the publication of data and subsequent impact on clinical practice for the use of total neoadjuvant therapy for rectal cancer.

### Study population

**FIGURE 1:**
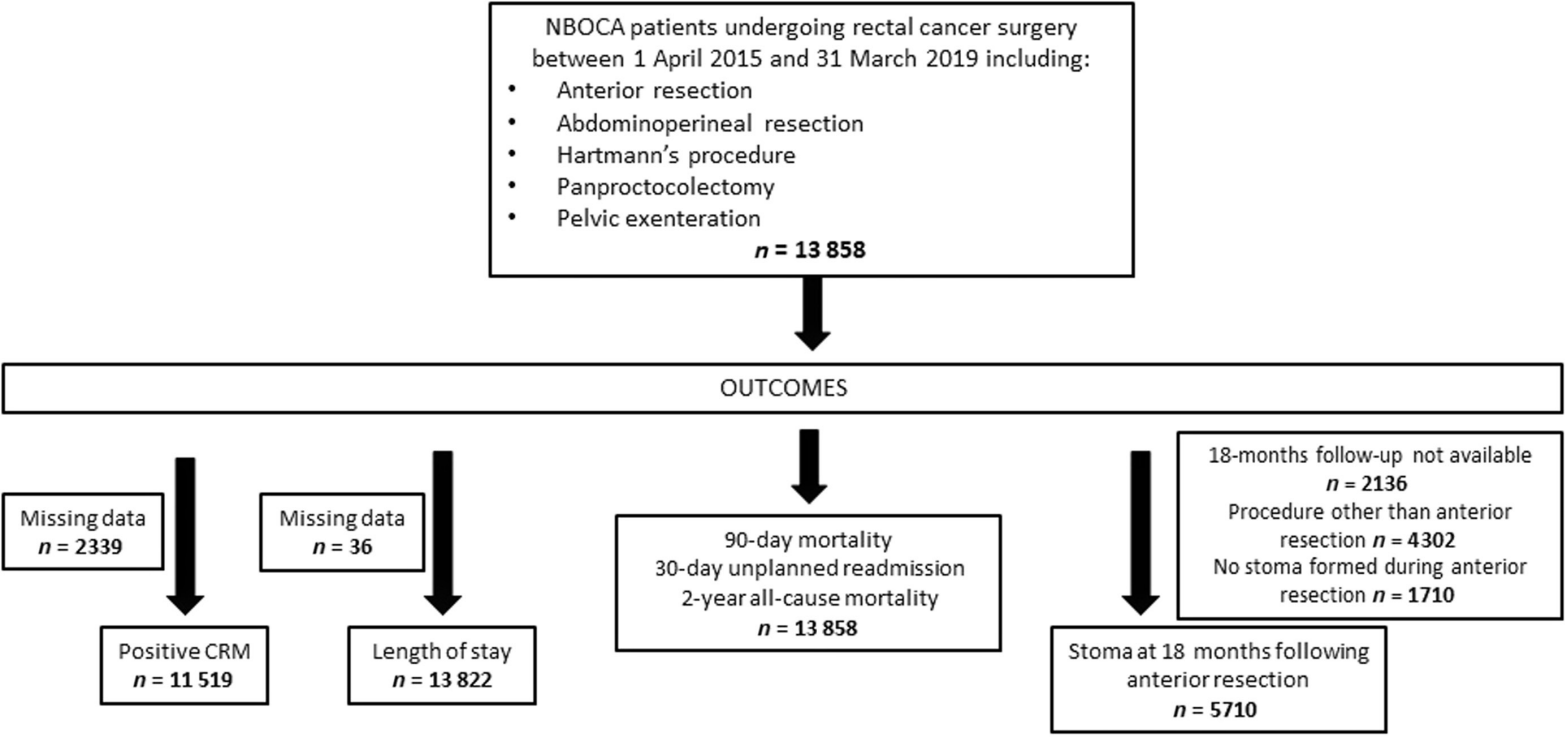
Flowchart for patients included in the study (CRM, circumferential resection margin; NBOCA, National Bowel Cancer Audit).

Patients undergoing a major resection for rectal cancer, excluding those not treated, treated with nonoperative palliative intent, or treated with organ preservation intention (transanal techniques, local excisions or complete clinical responders on a watch‐and‐wait protocol) between 1 April 2015 and 31 March 2019 according to the NBOCA were identified (Figure 1). Procedures included were anterior resection, abdominoperineal resection (APR), low Hartmann's procedure, panproctocolectomy, and pelvic exenteration.

### Hospital‐level volumes

Using previously developed methodology, mean annual hospital‐level volumes were calculated from HES, in order to maximize the capture of procedures [24]. ‘Hospital’ refers to individual English NHS hospital sites performing rectal cancer surgery (multiple hospital sites can make up a NHS trust, the organizational unit within the English NHS). All 166 hospital sites performed rectal cancer surgery across all years of the included time frame.

### Surgeon‐level volumes

Similarly, using previously developed methodology, mean annual surgeon‐level volumes were calculated [24]. This made use of NBOCA, HES and General Medical Council (GMC) data to maximize the capture of procedures and restrict surgeon‐level analyses to active general surgeons. For records where there was a discrepancy between NBOCA and HES on the responsible surgeon, the information recorded in NBOCA was deemed to be the most accurate, because NBOCA data are used for the NHS Clinical Outcomes publication scheme (individual surgeon outcomes are published in the public domain) and therefore routinely scrutinized on an annual basis [25].

The mean annual volume was calculated as the number of rectal cancer procedures performed during the surgeon's active period divided by the duration of the active period. The duration of the active period was defined as the number of financial years in which the surgeon had rectal cancer procedures recorded.

### Outcomes

#### Ninety‐day mortality

Patient records linked to ONS mortality data were used to ascertain patients who died within 90 days of their rectal cancer surgery.

#### Thirty‐day unplanned readmission

HES records were used to identify any unplanned admission for any cause and to any English NHS hospital within 30 days of the date of discharge.

#### Thirty‐day unplanned return to theatre

HES records were used to identify patients who returned to theatre following their primary rectal cancer surgery using a preexisting validated coding algorithm based on Office of Population Censuses and Surveys Classification of Surgical Operations and Procedures, 4th revision (OPCS‐4) codes [26].

#### Stoma at 18 months following anterior resection

This was defined as the proportion of patients who still had a stoma 18 months after an anterior resection for rectal cancer. The vast majority of stomas formed during an anterior resection are temporary and are expected to be reversed. Within the English NHS, this is a reported national outcome measure which is considered reflective of the quality of complex decision‐making, postoperative complications such as anastomotic leak and progression of disease, as well as differences in the prioritization of stoma reversals and associated pathways.

OPCS‐4 codes within HES records (G753 or H154) were used to identify patients undergoing reversal procedures. Patients needed to have at least 18 months follow‐up after their rectal cancer surgery to be included within this analysis (this includes patients undergoing major resection until 30 September 2018).

#### Positive circumferential resection margin

NBOCA records provided information on circumferential resection margin (CRM) status, which reflects a key determinant of the quality of rectal cancer surgery. A positive CRM is defined as a resection margin which is less than 1 mm from the tumour (R1) or where visible tumour remains in situ (R2).

#### Length of stay

HES records were used to calculate the length of inpatient stay from the date of rectal cancer surgery. A binary outcome was generated based on whether the hospital stay was greater than 14 days in order to try to capture those patients with a significant delay in postoperative recovery, probably due to immediate complications.

#### Two‐year all‐cause mortality rate

NBOCA and HES records linked to ONS mortality records were used to identify patients who died within 2 years of the date of rectal cancer surgery from any cause. Follow‐up time was censored at 16 April 2020 or 2 years, whichever was earliest. Approximately two‐thirds of patients were recorded in ONS as dying from colorectal cancer.

### Statistical analysis

Patient, tumour and treatment characteristics were compared using chi‐square tests according to tertiles of mean annual hospital and surgeon volumes.

Multilevel multivariable logistic regression was used to model a continuous relationship between each binary outcome and hospital and surgeon volumes, using a linear plus a quadratic term for volume. A random intercept at hospital or surgeon level was used to account for clustering. For each outcome a global Wald test was used to evaluate the statistical significance of the linear and quadratic terms for volume together.

Several sensitivity analyses were performed. This included modelling volume as a categorical variable (according to tertiles and quintiles) and also estimating models one‐by‐one excluding emergency patients, those having pelvic exenteration, and those having robotic surgery.

Risk adjustment was undertaken for age, sex, socioeconomic status according to the index of multiple deprivation quintiles, Royal College of Surgeons Charlson comorbidity score, ECOG performance status, ASA grade, surgical urgency (emergency/urgent versus elective/scheduled), surgical procedure (pelvic exenteration versus other procedure), pathological TNM staging and radiotherapy use (long‐course, short‐course or none). The choice of risk factors included was guided by the National Institute for Health and Care Excellence's review of evidence, as well as reflecting patient case mix and complexity of the case rather than choices made about the surgical approach and procedure [11].

To avoid ‘over‐fitting’ and allow a wide range of plausibly shaped relationships to be modelled (most likely linear or monotonic relationships with a greater or lesser degree of curvature), we decided a priori to model a linear plus quadratic relationship [27]. The adequacy of a linear plus quadratic relationship between volume and each binary outcome was assessed by superimposing the fitted line onto a graph of the predicted risk‐adjusted outcome with 95% confidence intervals, in six equally sized categories of volume, setting the value of all other covariates to the mean value. Patients with missing data on outcomes (CRM status and length of stay) were excluded from the analyses (Figure 1).

Of the 13 858 patients included in the analysis, 3249 (23%) had missing data for at least one of the data items included in the risk‐adjustment models. Of these 3249 patients, 1918 (59%) had only one data item missing. Missing data were assumed to be missing at random based on assessment of the characteristics of those patients with and without missing data.

Missing values for risk‐adjustment variables were imputed with multiple imputation using chained equations, creating 20 datasets and using Rubin's rules to combine the estimated odds ratios across the datasets [28]. The imputation model used logistic regression for binary variables and multinomial regression for categorical variables with more than two categories. The imputation model included all variables within the analysis model (including all outcome variables) and any additional variables thought to predict ‘missingness’.

All statistical analyses were undertaken using Stata version 15.

## RESULTS

### Hospital‐ and surgeon‐level volumes

A total of 13 858 patients undergoing rectal cancer surgery at 166 English NHS hospital sites between 1 April 2015 and 31 March 2019 were included in the hospital volume analyses, and 13 841 patients were included in the surgeon analyses with 846 active general surgeons. Seventeen patients were excluded because the responsible surgeon could not be identified.

At hospital level, the median annual number of procedures was 26 [interquartile range (IQR) 19–36, range 1–74]. Six hospitals (3.6%) performed fewer than 10 resections per year and 43 hospitals (25.9%) performed fewer than 20 resections per year. At surgeon level, the median annual number of procedures was 5 (IQR 3–7, range 1–31). Seventy five surgeons (8.9%) performed only one resection, 381 (45.0%) performed fewer than five resections and 756 (89.3%) performed fewer than 10 resections per year.

High‐volume surgeons were more likely to work in high‐volume hospitals (*p* < 0.001), although 13% (33/256) of high‐volume surgeons worked in the lowest‐volume hospitals and 34% (91/268) of low‐volume surgeons worked in the highest‐volume hospitals (Table S1).

### Patient characteristics according to hospital‐ and surgeon‐level volumes

Both high‐volume hospitals and high‐volume surgeons were less likely than low‐volume hospitals and low‐volume surgeons to treat ethnic minority groups and more likely to treat affluent patients (Table 1). High‐volume hospitals were less likely to perform sphincter‐sparing procedures and more likely to perform robotic surgery. High‐volume surgeons were more likely to perform elective surgery, sphincter‐sparing procedures and robotic surgery. Pelvic exenterations were uncommon but tended to be performed by high‐volume surgeons in high‐volume hospitals.

**TABLE 1:** Characteristics according to tertiles of hospital‐ and surgeon‐level mean annual volumes.

	Hospital‐level volume				Surgeon‐level volume			
	Low volume (<22; 60 hospitals), *n* (%) (*N* = 2701)	Mid volume (22–31; 51 hospitals), *n* (%) (*N* = 4066)	High volume (32–74; 55 hospitals), *n* (%) (*N* = 7091)	*p*‐value	Low volume (1–3; 268 surgeons), *n* (%) (*N* = 1414)	Mid volume (4–6; 322 surgeons), *n* (%) (*N* = 4747)	High volume (>6, 256 surgeons), *n* (%) (*N* = 7680)	*p*‐value
Age (years)								
<50	225 (8.3)	273 (6.7)	515 (7.3)	0.021*	98 (6.9)	354 (7.5)	558 (7.3)	0.265
50–59	512 (19.0)	726 (17.9)	1265 (17.8)		237 (16.8)	831 (17.5)	1432 (18.6)	
60–74	1259 (46.6)	2091 (51.4)	3528 (49.8)		740 (52.3)	2380 (50.1)	3753 (48.9)	
75–84	622 (23.0)	857 (21.1)	1574 (22.2)		291 (20.6)	1048 (22.1)	1710 (22.3)	
≥85	83 (3.1)	119 (2.9)	209 (2.9)		48 (3.4)	134 (2.8)	227 (3.0)	
Sex								
Male	1787 (66.2)	2639 (64.9)	4599 (64.9)	0.452	948 (67.0)	3041 (64.1)	5026 (65.4)	0.082
Female	914 (33.8)	1427 (35.1)	2492 (35.1)		466 (33.0)	1706 (35.9)	2654 (34.6)	
Ethnicity								
White	2383 (92.1)	3709 (95.8)	6418 (95.8)	<0.001*	1253 (92.2)	4328 (95.3)	6913 (95.5)	<0.001*
Other	205 (7.9)	161 (4.2)	280 (4.2)		106 (7.8)	214 (4.7)	326 (4.5)	
Missing	113 (4.2)	196 (4.8)	393 (5.5)		55 (3.9)	205 (4.3)	441 (5.7)	
IMDQ								
1 (most deprived)	411 (15.2)	697 (17.2)	977 (13.8)	<0.001*	241 (17.1)	771 (16.3)	1069 (13.9)	<0.001*
2	543 (20.1)	725 (17.9)	1164 (16.4)		262 (18.5)	861 (18.2)	1304 (17.0)	
3	603 (22.4)	856 (21.1)	1501 (21.2)		298 (21.1)	1016 (21.4)	1644 (21.5)	
4	601 (22.3)	913 (22.5)	1631 (23.0)		326 (23.1)	1028 (21.7)	1788 (23.3)	
5 (least deprived)	539 (20.0)	864 (21.3)	1806 (25.5)		286 (20.2)	1061 (22.4)	1859 (24.3)	
Missing	4 (0.1)	11 (0.3)	12 (0.2)		1 (0.1)	10 (0.2)	16 (0.2)	
RCS Charlson score								
0	1543 (57.1)	2366 (58.2)	4243 (59.8)	0.004*	805 (56.9)	2771 (58.4)	4570 (59.5)	0.357
1	829 (30.7)	1132 (27.8)	1948 (27.5)		416 (29.4)	1365 (28.8)	2122 (27.6)	
≥2	329 (12.2)	568 (14.0)	900 (12.7)		193 (13.6)	611 (12.9)	988 (12.9)	
Performance status								
0	1550 (60.6)	2370 (65.2)	3884 (64.9)	<0.001*	804 (62.5)	2635 (62.6)	4362 (65.4)	0.002*
1	732 (28.6)	1026 (28.2)	1608 (26.9)		350 (27.2)	1229 (29.2)	1779 (26.7)	
≥2	276 (10.8)	237 (6.5)	495 (8.3)		133 (10.3)	343 (8.2)	529 (7.9)	
Missing	143 (5.3)	433 (10.6)	1104 (15.6)		127 (9.0)	540 (11.4)	1010 (13.2)	
ASA grade								
1	405 (15.7)	597 (15.5)	1004 (14.9)	0.04*	209 (15.5)	708 (15.6)	1087 (15.0)	0.893
2	1524 (59.0)	2408 (62.3)	4128 (61.3)		816 (60.5)	2768 (60.8)	4468 (61.5)	
≥3	652 (25.3)	859 (22.2)	1606 (23.8)		323 (24.0)	1074 (23.6)	1713 (23.6)	
Missing	120 (4.4)	202 (5.0)	353 (5.0)					
Surgical access								
Open	580 (21.6)	1058 (26.1)	1952 (27.6)	<0.001*	430 (30.6)	1500 (31.7)	1646 (21.5)	<0.001
Laparoscopic	2052 (76.3)	2758 (68.0)	4654 (65.8)		959 (68.2)	3110 (65.7)	5392 (70.4)	
Robotic	56 (2.1)	238 (5.9)	463 (6.5)		18 (1.3)	122 (2.6)	617 (8.1)	
Missing	13 (0.5)	12 (0.3)	22 (0.3)					
Surgical urgency								
Elective/scheduled	2582 (95.8)	3858 (95.3)	6894 (97.6)	<0.001*	1307 (92.7)	4574 (96.6)	7447 (97.4)	<0.001*
Emergency/urgent	113 (4.2)	191 (4.7)	173 (2.5)		103 (7.3)	161 (3.4)	202 (2.6)	
Missing	6 (0.2)	17 (0.4)	24 (0.3)		4 (0.3)	12 (0.3)	31 (0.4)	
Surgical procedure								
Anterior resection	1759 (65.1)	2554 (62.8)	4468 (63.0)	<0.001*	883 (62.4)	2977 (62.7)	4917 (64.0)	<0.001*
APR	629 (23.3)	1038 (25.5)	1778 (25.1)		315 (22.3)	1202 (25.3)	1925 (25.1)	
Hartmann's	279 (10.3)	402 (9.9)	663 (9.3)		181 (12.8)	490 (10.3)	664 (8.6)	
Pelvic exenteration	4 (0.1)	22 (0.5)	88 (1.2)		8 (0.6)	16 (0.3)	90 (1.2)	
Panproctocolectomy	30 (1.1)	50 (1.2)	94 (1.3)		27 (1.9)	62 (1.3)	84 (1.1)	
Pathological T‐stage								
T1	320 (12.7)	465 (12.3)	914 (14.0)	0.211	140 (10.8)	582 (13.1)	976 (13.8)	0.05
T2	715 (28.5)	1130 (29.8)	1866 (28.5)		361 (27.7)	1308 (29.4)	2038 (28.8)	
T3	1278 (50.9)	1910 (50.4)	3259 (49.8)		689 (53.0)	2213 (49.8)	3543 (50.0)	
T4	200 (8.0)	282 (7.4)	502 (7.7)		111 (8.5)	340 (7.7)	526 (7.4)	
Missing	188 (7.0)	279 (6.9)	550 (7.8)		113 (8.0)	304 (6.4)	597 (7.8)	
Pathological N‐stage								
N0	1592 (63.7)	2407 (63.4)	4168 (63.7)	0.231	825 (63.5)	2818 (63.5)	4515 (63.7)	0.002*
N1	657 (26.3)	942 (24.8)	1632 (25.0)		335 (25.8)	1120 (25.3)	1773 (25.0)	
N2	251 (10.0)	445 (11.7)	741 (11.3)		139 (10.7)	497 (11.2)	799 (11.3)	
Missing	201 (7.4)	272 (6.7)	550 (7.8)		115 (8.1)	312 (6.6)	593 (7.7)	
Pathological M‐stage								
M0	2344 (95.5)	3612 (96.4)	6041 (96.1)	0.151	1198 (94.6)	4160 (95.9)	6628 (96.5)	0.002*
M1	111 (4.5)	133 (3.6)	242 (3.9)		69 (5.4)	179 (4.1)	237 (3.5)	
Missing	246 (9.1)	321 (7.9)	808 (11.4)		147 (10.4)	408 (8.6)	815 (10.6)	
Radiotherapy								
No radiotherapy	1750 (64.8)	2670 (65.7)	4752 (67.0)	0.013*	957 (67.7)	3097 (65.2)	5105 (66.5)	<0.001*
Long course	710 (26.3)	1081 (26.6)	1849 (26.1)		338 (23.9)	1246 (26.2)	2053 (26.7)	
Short course	241 (8.9)	315 (7.7)	490 (6.9)		119 (8.4)	404 (8.5)	522 (6.8)	

Abbreviations: APR, abdominoperineal resection; ASA, American Society of Anesthesologists; IMDQ, index of multiple deprivation quintiles; RCS, Royal College of Surgeons.

*Statistically significant *p*‐value <0.05.

Although statistically significant, many of the other observed differences in characteristics at hospital and surgeon level are unlikely to be clinically significant.

### Outcomes

Considering all 13 858 included patients, we found that 253 (1.8%) died within 90 days of their rectal cancer resection, 1920 (13.9%) had an unplanned 30‐day readmission, 1595 (11.5%) had a 30‐day unplanned return to theatre, and 1303 (9.4%) died within 2 years from any cause.

Of the 5710 patients who underwent an anterior resection with a stoma and had sufficient follow‐up, 2051 (35.9%) still had a stoma at 18 months. Of the 11 519 patients with CRM information, 1021 (8.9%) had positive margins. Of the 13 822 patients with length of stay information, 2941 (21.3%) had a stay of longer than 14 days.

### Volume–outcome relationship

**FIGURE 2:**
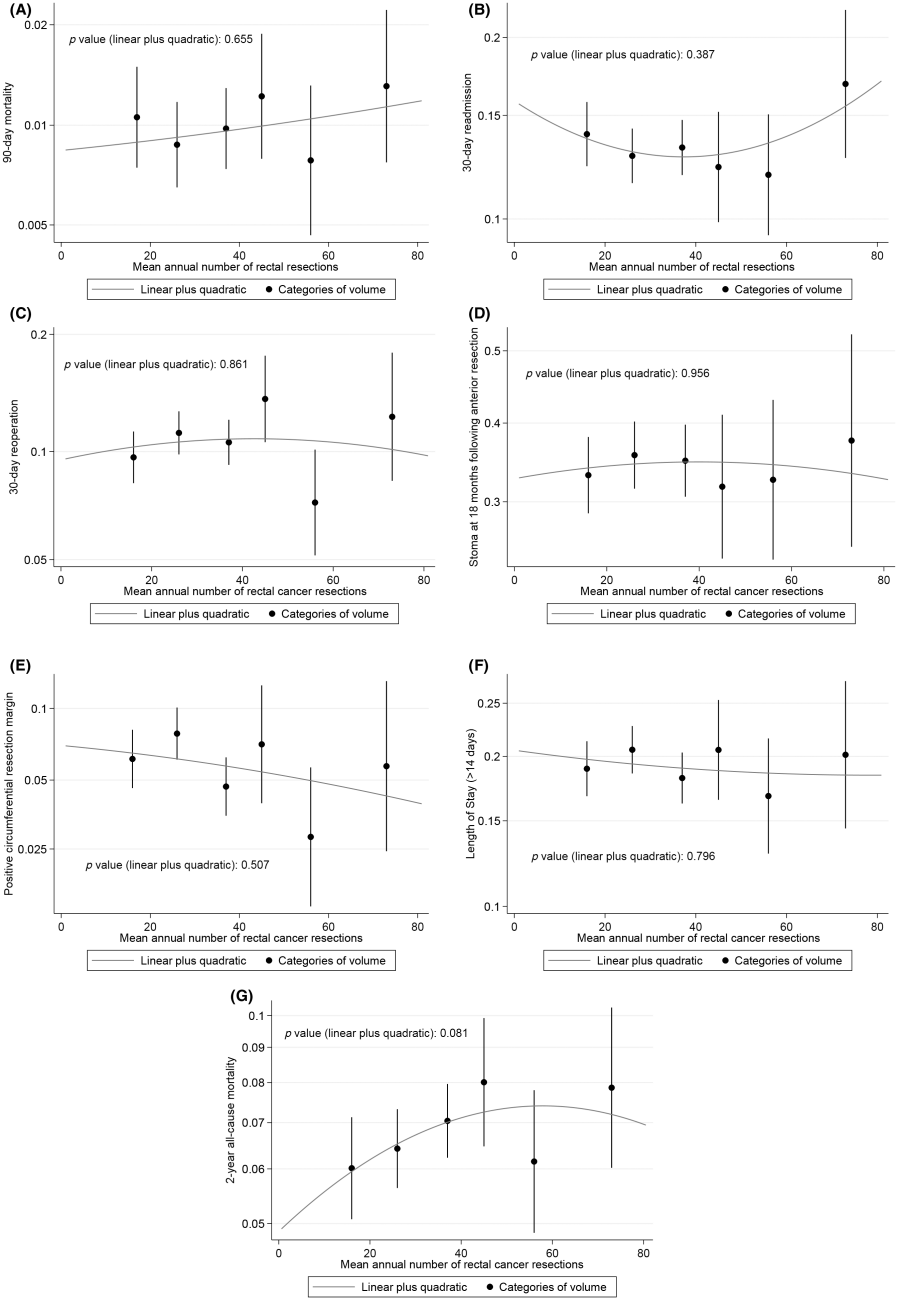
Linear–quadratic graphs showing the risk‐adjusted volume–outcome relationship at hospital level for: (A) 90‐day mortality; (B) 30‐day unplanned readmission; (C) 30‐day unplanned return to theatre; (D) stoma at 18 months following anterior resection; (E) positive circumferential resection margin; (F) prolonged length of stay (>14 days); and (G) 2‐year all‐cause mortality. The *y*‐axis is plotted on a risk scale for all graphs.

**FIGURE 3:**
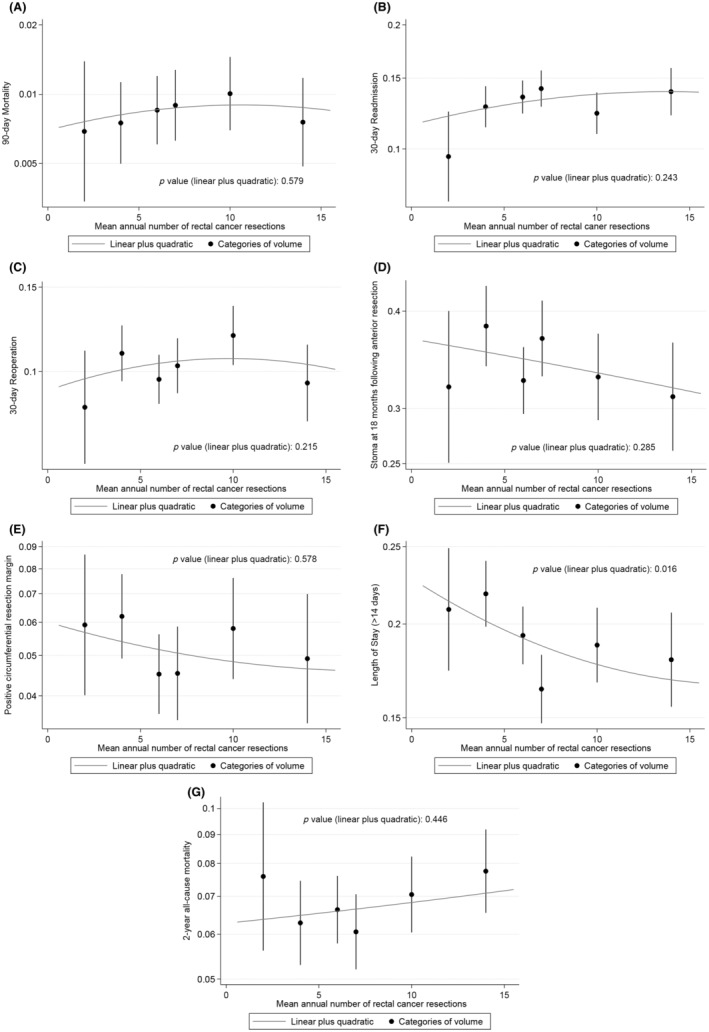
Linear–quadratic graphs showing the risk‐adjusted volume–outcome relationship at surgeon level for: (A) 90‐day mortality; (B) 30‐day unplanned readmission; (C) 30‐day unplanned return to theatre; (D) stoma at 18 months following anterior resection; (E) positive circumferential resection margin; (F) prolonged length of stay (>14 days); and (G) 2‐year all‐cause mortality. The y‐axis is plotted on a risk scale for all graphs.

After risk‐adjustment, the linear–quadratic relationship between volume and outcome was a good fit to the data for each outcome (Figures 2 and 3). With risk adjustment there was no statistically significant volume–outcome relationship at hospital level for the linear and quadratic terms for any outcomes including 90‐day mortality (*p* = 0.655), 30‐day unplanned readmission (*p* = 0.387), unplanned return to theatre (*p* = 0.861), stoma at 18 months following anterior resection (*p* = 0.956), positive CRM (*p* = 0.507), length of stay (*p* = 0.796) and 2‐year all‐cause mortality rate (*p* = 0.137; Figure 2, Table S2).

High‐volume surgeons had a significantly shorter risk‐adjusted length of stay than low‐volume surgeons (*p* = 0.016; Figure 3). There was no statistically significant risk‐adjusted volume–outcome relationship at surgeon level for any other outcomes including 90‐day mortality (*p* = 0.579), 30‐day unplanned readmission (*p* = 0.243), unplanned return to theatre (*p* = 0.215), stoma at 18 months following anterior resection (*p* = 0.285), positive CRM (*p* = 0.578) and 2‐year all‐cause mortality rate (*p* = 0.535; Figure 3, Table S2).

Sensitivity analyses performed to model volume as a categorical variable (tertiles and quintiles) did not significantly change the results (Table S3). Similarly, sensitivity analyses modelling outcomes excluding emergency patients, those having pelvic exenterations and those having robotic procedures did not significantly change the results (Table S4).

## DISCUSSION

This large national study explores the relationship between hospital and surgeon rectal cancer resection volumes and a comprehensive set of outcome measures available from routinely collected data within the English NHS. It demonstrates that almost half of surgeons do not meet the recently recommended national quality standard of carrying out at least five major rectal cancer resections per year [14]. Within this study, a volume–outcome relationship was demonstrated for reduced length of stay for high‐volume surgeons. However, we did not demonstrate a volume–outcome relationship for any of the other outcomes (including postoperative mortality, complications and resection margins) at hospital or surgeon level.

### Limitations and strengths

The main limitation of this study is that, although national high‐quality data were used, the data were not captured for the purpose of evaluating a volume–outcome relationship. However, case ascertainment is high and validation of surgeon information was undertaken using two data sources with 92% agreement on surgeon‐level information, and further verification through checks against the GMC register of medical practitioners within the United Kingdom [24]. The datasets only capture the ‘responsible’ senior surgeon and this may not always be the surgeon who carried out the actual procedure, which may attenuate our findings (e.g. a surgeon in training performing a procedure under supervision by a consultant).

Also, it was not possible to capture the full complexity of each procedure from the available data. Distance of the tumour from the anal verge and body mass index are captured but are currently too poorly completed within the available national datasets to be included in any risk‐adjustment analysis. A previous study found that high‐volume surgeons tended to remove lower tumours that require more complex surgery, which would lead to an underestimation of the volume–outcome relationship [29]. However, for the 6947 patients (50.1%) for whom this information was available in our study there was little association between volume and tumour height. It therefore seems unlikely that risk adjustment for tumour height would have significantly altered the findings.

There are some other measures that would have been important to adjust for, but these were either poorly completed or not captured within the available data. These include histological features (e.g. lymphovascular invasion, tumour budding and differentiation), genomics (e.g. microsatellite instability status), preoperative MRI results, which would have allowed an assessment of the appropriateness of neoadjuvant therapy, and details about the MDT (e.g. nature, composition and frequency of meetings).

Finally, some of the measures used (e.g. unplanned return to theatre) are more difficult to define within routinely collected data or are surrogate markers. For example, 2‐year all‐cause mortality was used due to restricted follow‐up and because locoregional recurrence is not captured in routine data sources, and it may not necessarily reflect longer‐term disease‐free or overall survival outcomes [30]. Ideally, more oncological outcomes would have been evaluated including, for example, high‐quality TME surgery and lymph node yield.

There are several strengths of this study. First, contrary to most of the other studies in this area, volume was modelled as a continuous variable to overcome the limitations associated with arbitrary cut‐offs, which make the interpretation and pooling of results across studies problematic [31]. Second, multilevel modelling was used to account for the clustering of outcomes in hospitals and surgeons. Ideally, we wanted to simultaneously account for the clustering of patients within surgeons, and the clustering of surgeons within hospitals, but the results did not converge with multiply imputed data. Fourth, comprehensive risk adjustment was undertaken in line with national recommendations [32]. Finally, contemporary data are reflective of the current practice (e.g. minimal access surgery) [11].

### Interpretation of findings

Reduced length of stay for high‐volume surgeons has been found previously, although some of these earlier studies also included colon cancer patients [12, 33]. This finding may be explained by high‐volume surgeons having increased access to laparoscopic and robotic techniques which are associated with a faster recovery [34]. This interpretation is supported by the loss of statistical significance when surgical access was added to the risk‐adjustment model (results not shown). High‐volume surgeons may be more likely to engage with Enhanced Recovery After Surgery (ERAS) programmes [5].

To date, the evidence for a volume–outcome relationship in rectal cancer surgery has been conflicting, although studies demonstrating a relationship always suggest better outcomes in high‐volume hospitals and surgeons. Studies showing a relationship between high hospital and surgeon volumes and overall survival, CRM rates, perioperative complications, local recurrence, permanent stoma rate and perioperative mortality have been identified, although there are equal numbers of studies that fail to provide statistically significant evidence of a relationship [11, 31].

There are several explanations why we did not demonstrate a volume–outcome relationship. Although we performed extensive risk‐adjustment, it is probable that we have not fully adjusted for the complexity of surgery or patient selection and there is residual confounding. It is possible that high‐volume hospitals and surgeons are operating on more complex cases due to preexisting referral pathways [14]. These same high‐volume providers may practise less risk averse behaviour due to their experience, as well as accepting referrals for patients with higher baseline morbidity requiring specialist expertise in the perioperative phase (e.g. patients requiring access to renal dialysis). These factors have the potential to dilute any volume–outcome relationship that may be observed.

Within the United States, the Leapfrog Group have made an annual minimum recommendation of six rectal cancer resections per surgeon [35]. In this study, 70% of surgeons do not meet this threshold and there is no clear evidence of centralization of high‐volume surgeons within high‐volume hospitals. It may be that surgeon volumes are too low to see an association between volume and outcomes. It is also difficult to measure the quality of shared decision‐making and patient selection within the MDT setting from the routinely collected national datasets. For example, whether appropriate decisions have been made regarding suitability for oncological therapies, or watch‐and‐wait strategies [36]. Some available outcomes are more likely to be affected by the overall MDT recommendations, rather than the individual surgeon's performance; 2‐year all‐cause mortality rate is a good example, with variation in adjuvant chemotherapy potentially being a contributory factor [37].

Some of the analyses are likely to be underpowered due to low event rates, particularly the surgeon‐level analyses. Although our national study has not demonstrated a volume–outcome association, it may be that a study including an even larger group of patients would provide evidence of a relationship. Collecting specific data on outcomes and key performance indicators (e.g. unplanned return to theatre) may help to identify a volume–outcome association. In addition, we are not currently able to capture patient‐reported measures such as bowel, urinary and sexual function, which are critical measures for survivorship [38].

Finally, these results might simply reflect a true lack of volume–outcome relationship. We might expect to see a volume–outcome relationship for rectal cancer surgery as it represents complex oncological surgery for which volume–outcome relationships are evident in other specialities [7]. However, one study demonstrated wide variation in elective colorectal surgery outcomes in the English NHS, even amongst the very highest‐volume surgeons (e.g. mortality rates ranging from 0% to 7.7%) [12]. Additional studies have also demonstrated variation in outcomes across the whole volume spectrum [39, 40].

It may be that adherence to a range of evidence‐based process measures, such as those defined within the United States in the National Accreditation Program for Rectal Cancer (NAPRC), is the real determinant in improving standards and therefore outcomes, rather than volume per se [41]. The NAPRC promotes standards but does not recommend minimum surgical volumes. Similarly in the UK, national guidelines recommend, for example, offering neoadjuvant therapy to patients with Stage II/III rectal cancer [14].

### Implications

Volume‐based policies are guided by assumptions that increased patient volumes enable greater specialization of all healthcare professionals contributing to the patient's care, and greater experience and expertise: the ‘practice makes perfect’ hypothesis [42]. However, the current study shows no evidence for a volume–outcome relationship and suggests that the centralization of rectal cancer services is likely to be more effective if it is based on factors that are known to improve quality rather than on operative volumes alone. As demonstrated previously, operative volume appears to be an imperfect proxy for the quality of rectal cancer care [35].

A recent study from Ireland, including patients undergoing curative major resection for Stage I–III rectal cancer, showed temporal improvements following centralization [43]. It focused on survival outcomes and process measures (e.g. choice of surgical procedure and use of neoadjuvant and adjuvant therapies). Although it can be assumed that operative volumes increased as a result of the centralization, there was no quantification or direct exploration of the volume–outcome relationship. The study hypothesized that ‘better surgery’ (defined as TME quality, R0 resection rate and high nodal yield), ‘expert specialist input’ within specialist MDTs and the ‘definition and monitoring of key performance indicators’ may have led to improvements [43]. This supports our findings that factors beyond operative volume alone need to be considered in order to improve rectal cancer outcomes.

Similarly, a study evaluating the centralization of oesophago‐gastric cancer services in the UK found temporal improvements in postoperative mortality but was unable to attribute this to increases in operative volume alone [7]. It hypothesized that improvements in medical care (e.g. oncological treatments and staging) and increases in cancer‐related operative training opportunities may contribute.

The potential benefits of centralization include: better institutional structure, more streamlined care processes, more frequent use of protocols supporting decision‐making, wider availability of advanced and innovative surgical techniques (e.g. liver resections [44] and hyperthermic intraperitoneal chemotherapy), greater use of active salvage in the event of postoperative complications, improved training opportunities and increased efficiency due to optimization of resource utilization.

The main disadvantages include additional travel distances for patients [45], and capacity problems. A recent health services planning model has demonstrated the potential impacts of five different centralization scenarios for rectal cancer services and might facilitate more informed discussions [46].

## CONCLUSIONS

This study has provided additional evidence regarding the volume–outcome relationship in rectal cancer surgery within the English NHS. We found that a substantial number of surgeons are not currently performing the national recommended annual volume of rectal cancer resections. However, apart from a reduced length of stay for higher‐volume surgeons, we were not able to demonstrate significant volume–outcome relationships within this study at hospital or surgeon level.

Given that current evidence is lacking for increasing operative volume in isolation, all essential aspects of high‐quality care should be balanced in the event of centralization of multidisciplinary rectal cancer services. A wide range of evidence‐based process measures across the whole care pathway, including those important to patients, should be evaluated to enhance rectal cancer surgery outcomes and ensure that patients are appropriately directed.

## AUTHOR CONTRIBUTIONS

**Jemma M. Boyle:** Conceptualization; investigation; writing – original draft; methodology; writing – review and editing; validation; visualization; formal analysis; data curation. **Jan van der Meulen:** Writing – review and editing; methodology; supervision. **Angela Kuryba:** Writing – review and editing; data curation; methodology. **Thomas E. Cowling:** Writing – review and editing; methodology; supervision. **Michael S. Braun:** Writing – review and editing; supervision. **Ajay Aggarwal:** Writing – review and editing; supervision. **Kate Walker:** Methodology; writing – review and editing; supervision; conceptualization. **Nicola S. Fearnhead:** Conceptualization; writing – review and editing; supervision.

## FUNDING INFORMATION

AA was supported by a National Institute for Health Research (NIHR) Advanced Fellowship [NIHR300599] and TEC by a fellowship from the Medical Research Council [MR/S020470/1]. For the remaining authors none were declared.

## CONFLICT OF INTEREST STATEMENT

None to declare.

## ETHICS STATEMENT

Not required.

## Supporting information


Appendix S1

## Data Availability

The data that support the findings of this study are available from HQIP. Restrictions apply to the availability of these data, which were used under license for this study. Data are available from https://www.hqip.org.uk/national‐programmes/accessing‐ncapop‐data/#.Y‐ZRBK3P1hE with the permission of HQIP.

## References

[1] Begg CB , Cramer LD , Hoskins WJ , Brennan MF . Impact of hospital volume on operative mortality for major cancer surgery. JAMA. 1998;280(20):1747–1751. 9842949 10.1001/jama.280.20.1747

[2] Finlayson EV , Goodney PP , Birkmeyer JD . Hospital volume and operative mortality in cancer surgery. A national study. Arch Surg. 2003;138(7):721–725. 12860752 10.1001/archsurg.138.7.721

[3] Birkmeyer JD , Stukel TA , Siewers AE , Goodney PP , Wennberg DE , Lucas FL . Surgeon volume and operative mortality in the United States. N Engl J Med. 2003;349(22):2117–2127. 14645640 10.1056/NEJMsa035205

[4] Luft HS , Bunker JP , Enthoven AC . Should operations be regionalized? The empirical relation between surgical volume and mortality. N Engl J Med. 1979;301(25):1364–1369. 503167 10.1056/NEJM197912203012503

[5] Ljungqvist O , Scott M , Fearon KC . Enhanced recovery after surgery: a review. JAMA Surg. 2017;152(3):292–298. 28097305 10.1001/jamasurg.2016.4952

[6] Vonlanthen R , Lodge P , Barkun JS , Farges O , Rogiers X , Soreide K , et al. Toward a consensus on centralization in surgery. Ann Surg. 2018;268(5):712–724. 30169394 10.1097/SLA.0000000000002965

[7] Varagunam M , Hardwick R , Riley S , Chadwick G , Cromwell DA , Groene O . Changes in volume, clinical practice and outcome after reorganisation of oesophago‐gastric cancer care in England: a longitudinal observational study. Eur J Surg Oncol. 2018;44(4):524–531. 29433991 10.1016/j.ejso.2018.01.001

[8] Salz T , Sandler RS . The effect of hospital and surgeon volume on outcomes for rectal cancer surgery. Clin Gastroenterol Hepatol. 2008;6(11):1185–1193. 18829393 10.1016/j.cgh.2008.05.023 PMC2582059

[9] Archampong D , Borowski D , Wille‐Jorgensen P , Iversen LH . Workload and surgeon's specialty for outcome after colorectal cancer surgery. Cochrane Database Syst Rev. 2012;(3):CD005391. 22419309 10.1002/14651858.CD005391.pub3 PMC12076000

[10] Chioreso C , Del Vecchio N , Schweizer ML , Schlichting J , Gribovskaja‐Rupp I , Charlton ME . Association between hospital and surgeon volume and rectal cancer surgery outcomes in patients with rectal cancer treated since 2000: systematic literature review and meta‐analysis. Dis Colon Rectum. 2018;61(11):1320–1332. 30286023 10.1097/DCR.0000000000001198 PMC7000208

[11] Rödel C , Graeven U , Fietkau R , Hohenberger W , Hothorn T , Arnold D , et al. Oxaliplatin added to fluorouracil‐based preoperative chemoradiotherapy and postoperative chemotherapy of locally advanced rectal cancer (the German CAO/ARO/AIO‐04 study): final results of the multicentre, open‐label, randomised, phase 3 trial. Lancet Oncol. 2015;16(8):979–989. 26189067 10.1016/S1470-2045(15)00159-X

[12] Burns EM , Bottle A , Almoudaris AM , Mamidanna R , Aylin P , Darzi A , et al. Hierarchical multilevel analysis of increased caseload volume and postoperative outcome after elective colorectal surgery. Br J Surg. 2013;100(11):1531–1538. 24037577 10.1002/bjs.9264

[13] Vallance AE , Fearnhead NS , Kuryba A , Hill J , Maxwell‐Armstrong C , Braun M , et al. Effect of public reporting of surgeons' outcomes on patient selection, ‘gaming’, and mortality in colorectal cancer surgery in England: population based cohort study. BMJ. 2018;361:k1581. 29720371 10.1136/bmj.k1581 PMC5930269

[14] National Institute for Health and Care Excellence . Colorectal cancer. NICE guideline 151 2020. Available from: https://www.nice.org.uk/guidance/ng151 32813481

[15] Link KH , Coy P , Roitman M , Link C , Kornmann M , Staib L . Minimum volume discussion in the treatment of colon and rectal cancer: a review of the current status and relevance of surgeon and hospital volume regarding result quality and the impact on health economics. Visc Med. 2017;33(2):140–147. 28560230 10.1159/000456044 PMC5447170

[16] National Bowel Cancer Audit . Available from: https://www.nboca.org.uk/

[17] Herbert A , Wijlaars L , Zylbersztejn A , Cromwell D , Hardelid P . Data resource profile: hospital episode statistics admitted patient care (HES APC). Int J Epidemiol. 2017;46(4):1093–1093i. 28338941 10.1093/ije/dyx015 PMC5837677

[18] CTRU Leeds Research Portal . FOxTROT 2 – a summary. Available from: https://ctru.leeds.ac.uk/foxtrot/for‐patients/about‐foxtrot‐2/

[19] Office for National Statistics . Deaths. Available from: https://www.ons.gov.uk/peoplepopulationandcommunity/birthsdeathsandmarriages/deaths

[20] National Bowel Cancer Audit Annual Report 2021. Available from: https://www.nboca.org.uk/content/uploads/2022/02/NBOCA‐2021‐AR‐Final.pdf

[21] Armitage JN , van der Meulen JH . Identifying co‐morbidity in surgical patients using administrative data with the Royal College of surgeons Charlson score. Br J Surg. 2010;97(5):772–781. 20306528 10.1002/bjs.6930

[22] The University of Manchester . Trial of wearable health technology for cancer patients opens. 2022. Available from: https://www.manchester.ac.uk/discover/news/trial‐of‐wearable‐health‐technology‐for‐cancer‐patients‐opens/

[23] Morris EJ , Finan PJ , Spencer K , Geh I , Crellin A , Quirke P , et al. Wide variation in the use of radiotherapy in the management of surgically treated rectal cancer across the English National Health Service. Clin Oncol. 2016;28(8):522–531. 10.1016/j.clon.2016.02.002 PMC4944647 26936609

[24] Nuttall M , van der Meulen J , Phillips N , Sharpin C , Gillatt D , McIntosh G , et al. A systematic review and critique of the literature relating hospital or surgeon volume to health outcomes for 3 urological cancer procedures. J Urol. 2004;172(6, Pt 1):2145–2152. 15538220 10.1097/01.ju.0000140257.05714.45

[25] Kizer KW . The volume–outcome conundrum. N Engl J Med. 2003;349(22):2159–2161. 14645645 10.1056/NEJMe038166

[26] Nicholson BD , Ordóñez‐Mena JM , Lay‐Flurrie S , Sheppard JP , Liyanage H , McGagh D , et al. Consultations for clinical features of possible cancer and associated urgent referrals before and during the COVID‐19 pandemic: an observational cohort study from English primary care. Br J Cancer. 2022;126(6):948–956. 34934176 10.1038/s41416-021-01666-6 PMC8691390

[27] Steyerberg EW , Eijkemans MJ , Habbema JD . Stepwise selection in small data sets: a simulation study of bias in logistic regression analysis. J Clin Epidemiol. 1999;52(10):935–942. 10513756 10.1016/s0895-4356(99)00103-1

[28] White IR , Royston P , Wood AM . Multiple imputation using chained equations: issues and guidance for practice. Stat Med. 2011;30(4):377–399. 21225900 10.1002/sim.4067

[29] Morris EJ , Birch R , West NP , Finan PJ , Forman D , Fairley L , et al. Low abdominoperineal excision rates are associated with high‐workload surgeons and lower tumour height. Is further specialization needed? Colorectal Dis. 2011;13(7):755–761. 20236155 10.1111/j.1463-1318.2010.02263.x

[30] van Gijn W , Gooiker GA , Wouters MW , Post PN , Tollenaar RA , van de Velde CJ . Volume and outcome in colorectal cancer surgery. Eur J Surg Oncol. 2010;36((Suppl 1):S55–S–63. 20615649 10.1016/j.ejso.2010.06.027

[31] Levaillant M , Marcilly R , Levaillant L , Michel P , Hamel‐Broza J‐F , Vallet B , et al. Assessing the hospital volume–outcome relationship in surgery: a scoping review. BMC Med Res Methodol. 2021;21(1):204. 34627143 10.1186/s12874-021-01396-6 PMC8502281

[32] National Institute for Health and Care Excellence . Colorectal cancer (update) [F1] surgical volumes and outcomes for rectal cancer. NICE Guideline NG151. 2020. Available from: https://www.nice.org.uk/guidance/ng151/evidence/f1‐surgical‐volumes‐and‐outcomes‐for‐rectal‐cancer‐pdf‐253058083705 32730010

[33] Borowski DW , Bradburn DM , Mills SJ , Bharathan B , Wilson RG , Ratcliffe AA , et al. Volume–outcome analysis of colorectal cancer‐related outcomes. Br J Surg. 2010;97(9):1416–1430. 20632311 10.1002/bjs.7111

[34] Schwenk W , Haase O , Neudecker J , Müller JM . Short term benefits for laparoscopic colorectal resection. Cochrane Database Syst Rev. 2005;(3):Cd003145. 16034888 10.1002/14651858.CD003145.pub2 PMC8693724

[35] Aquina CT , Becerra AZ , Fleming FJ , Cloyd JM , Tsung A , Pawlik TM , et al. Variation in outcomes across surgeons meeting the leapfrog volume standard for complex oncologic surgery. Cancer. 2021;127(21):4059–4071. 34292582 10.1002/cncr.33766

[36] Augestad KM , Lindsetmo RO , Stulberg J , Reynolds H , Senagore A , Champagne B , et al. International preoperative rectal cancer management: staging, neoadjuvant treatment, and impact of multidisciplinary teams. World J Surg. 2010;34(11):2689–2700. 20703471 10.1007/s00268-010-0738-3 PMC2949570

[37] Boyle JM , Kuryba A , Cowling TE , Aggarwal A , Hill J , van der Meulen J , et al. Determinants of variation in the use of adjuvant chemotherapy for stage III colon cancer in England. Clin Oncol. 2020;32(5):e135–e144. 10.1016/j.clon.2019.12.008 31926818

[38] Denlinger CS , Barsevick AM . The challenges of colorectal cancer survivorship. J Natl Compr Cancer Netw. 2009;7(8):883–894. 10.6004/jnccn.2009.0058 PMC3110673 19755048

[39] Burns EM , Bottle A , Aylin P , Darzi A , Nicholls RJ , Faiz O . Variation in reoperation after colorectal surgery in England as an indicator of surgical performance: retrospective analysis of hospital episode statistics. BMJ. 2011;343:d4836. 21846714 10.1136/bmj.d4836 PMC3156827

[40] Harrison A . Assessing the relationship between volume and outcome in hospital services: implications for service centralization. Health Serv Manage Res. 2012;25(1):1–6. 22323665 10.1258/hsmr.2011.011027

[41] Brady JT , Xu Z , Scarberry KB , Saad A , Fleming FJ , Remzi FH , et al. Evaluating the current status of rectal cancer care in the US: where we stand at the start of the commission on Cancer's National Accreditation Program for rectal cancer. J Am Coll Surg. 2018;226(5):881–890. 29580675 10.1016/j.jamcollsurg.2018.01.057

[42] Melnychuk M , Vindrola‐Padros C , Aitchison M , Clarke CS , Fulop NJ , Levermore C , et al. Centralising specialist cancer surgery services in England: survey of factors that matter to patients and carers and health professionals. BMC Cancer. 2018;18(1):226. 29486730 10.1186/s12885-018-4137-8 PMC6389051

[43] O'Connell E , McDevitt J , Hill ADK , McNamara DA , Burke JP . Centralisation of rectal cancer care has improved patient survival in the Republic of Ireland. Eur J Surg Oncol. 2022;48(4):890–895. 34774395 10.1016/j.ejso.2021.10.031

[44] Vallance AE , van der Meulen J , Kuryba A , Botterill ID , Hill J , Jayne DG , et al. Impact of hepatobiliary service centralization on treatment and outcomes in patients with colorectal cancer and liver metastases. Br J Surg. 2017;104(7):918–925. 28251644 10.1002/bjs.10501 PMC5484381

[45] Jones AP , Haynes R , Sauerzapf V , Crawford SM , Zhao H , Forman D . Travel time to hospital and treatment for breast, colon, rectum, lung, ovary and prostate cancer. Eur J Cancer. 2008;44(7):992–999. 18375117 10.1016/j.ejca.2008.02.001

[46] Aggarwal A , Han L , van der Geest S , Lewis D , Lievens Y , Borras J , et al. Health service planning to assess the expected impact of centralising specialist cancer services on travel times, equity, and outcomes: a national population‐based modelling study. Lancet Oncol. 2022;23:1211–1220. 35931090 10.1016/S1470-2045(22)00398-9

