# Supplementary material for: What is the impact of hospital and surgeon volumes on outcomes in rectal cancer surgery?
Source: Colorectal Dis. 2023 Sep 13;25(10):1981–93. doi: 10.1111/codi.16745 (PMC10946964; doi:10.1111/codi.16745)
Supplement: Supplementary file 1 — Appendix S1 [file CODI-25-1981-s001.docx]

**Supplemental content**

**Table S1** – Distribution of surgeon volumes (n=846), according to associated hospital volume.

| **Hospital Volume** | **Surgeon volume** | | |  |
| --- | --- | --- | --- | --- |
|  | **Low (1-3)**  **Column %** | **Middle (4-6)**  **Column %** | **High (>6)**  **Column %** | **P value (χ^2^)** |
| **Low (<22)** | 105 (39.2) | 86 (26.7) | 33 (12.9) | <0.001 |
| *Row %* | *(46.9)* | *(38.4)* | *(14.7)* |  |
| **Middle (22-31)** | 72 (26.9) | 113 (35.1) | 66 (25.8) |  |
| *Row %* | *(28.7)* | *(45.0)* | *(26.3)* |  |
| **High (32-74)** | 91 (34.0) | 123 (38.2) | 157 (61.3) |  |
| *Row %* | *(24.5)* | *(33.2)* | *(42.3)* |  |

**Table S2 –** Odds ratios with 95% confidence intervals for hospital and surgeon level volumes (linear term and quadratic term), from the risk-adjusted models for each individual outcome.

| **Hospital-level** | **Volume as a linear term**  **(2 decimal places)** | **Volume as a quadratic term**  **(6 decimal places)** |
| --- | --- | --- |
| 90-day mortality | 1.00 (0.97 to 1.04) | 1.000014 (0.999543 to 1.000485) |
| 30-day unplanned re-admission | 0.99 (0.97 to 1.01) | 1.000179 (0.999924 to 1.000433) |
| 30-day unplanned return theatre | 1.01 (0.98 to 1.03) | 0.9999196 (0.999583 to 1.000256) |
| Stoma at 18 months following anterior resection | 1.00 (0.97 to 1.04) | 0.9999409 (0.999528 to 1.000354) |
| Positive circumferential resection margin | 1.00 (0.95 to 1.04) | 0.9999612 (0.999349 to 1.000574) |
| Prolonged length of stay | 1.00 (0.98 to 1.02) | 1.000022 (0.999738 to 1.000306) |
| 2-year all-cause mortality | 1.01 (1.00 to 1.03) | 0.9998784 (0.999642 to 1.000115) |
|  |  |  |
| **Surgeon-level** |  |  |
| 90-day mortality | 1.05 (0.95 to 1.16) | 0.9977201 (0.993462 to 1.001997) |
| 30-day unplanned re-admission | 1.03 (0.99 to 1.07) | 0.9988549 (0.997291 to 1.000422) |
| 30-day unplanned return theatre | 1.04 (0.99 to 1.09) | 0.9981764 (0.996122 to 1.000235) |
| Stoma at 18 months following anterior resection | 0.99 (0.93 to 1.04) | 0.9998942 (0.997565 to 1.002229) |
| Positive circumferential resection margin | 0.97 (0.90 to 1.04) | 1.000902 (0.997445 to 1.004372) |
| Prolonged length of stay | 0.96 (0.93 to 1.00) | 1.001153 (0.999539 to 1.002770) |
| 2-year all-cause mortality | 1.01 (0.97 to 1.05) | 1.000071 (0.998484 to 1.001660) |
|  |  |  |

**Table S3** – Global *p* values for each risk-adjusted outcome using hospital and surgeon volume modelled as a categorical variable (tertiles or quintiles of volume).

*****denotes statistically significant p values

| **Hospital-level** | **Tertiles of volume** | **Quintiles of volume** |
| --- | --- | --- |
| 90-day mortality | 0.810 | 0.910 |
| 30-day unplanned re-admission | 0.796 | 0.915 |
| 30-day unplanned return theatre | 0.654 | 0.437 |
| Stoma at 18 months following anterior resection | 0.853 | 0.762 |
| Positive circumferential resection margin | 0.045*Ϯ | 0.126 |
| Prolonged length of stay | 0.536 | 0.671 |
| 2-year all-cause mortality | 0.061 | 0.171 |
|  |  |  |
| **Surgeon-level** |  |  |
| 90-day mortality | 0.677 | 0.576 |
| 30-day unplanned re-admission | 0.005*≠ | 0.112 |
| 30-day unplanned return theatre | 0.566 | 0.328 |
| Stoma at 18 months following anterior resection | 0.176 | 0.185 |
| Positive circumferential resection margin | 0.166 | 0.215 |
| Prolonged length of stay | <0.001* | 0.010* |
| 2-year all-cause mortality | 0.842 | 0.845 |
|  |  |  |

**Ϯ** Compared to the lowest hospital volume tertile (baseline), the OR for the middle hospital volume tertile was 1.31 (95% CI: 0.88 to 1.94) and for the highest hospital volume tertile was 0.81 (95% CI: 0.55 to 1.19).

≠ Compared to the lowest surgeon volume tertile (baseline), the OR for the middle surgeon volume tertile was 1.37 (95% CI: 1.12 to 1.66) and for the highest surgeon volume tertile was 1.34 (95% CI: 1.11 to 1.63).

**Table S4** – Global *p* values for each risk-adjusted outcome using hospital and surgeon volume modelled as a linear plus quadratic term with exclusion of emergency patients, patients undergoing pelvic exenteration surgery, and patients having robotic procedures.

*****denotes statistically significant p values

| **Hospital-level** | **Excluding emergency patients** | **Excluding patients having pelvic exenteration** | **Excluding patients having robotic surgery** |
| --- | --- | --- | --- |
| 90-day mortality | 0.428 | 0.598 | 0.477 |
| 30-day unplanned re-admission | 0.315 | 0.341 | 0.391 |
| 30-day unplanned return theatre | 0.839 | 0.758 | 0.885 |
| Stoma at 18 months following anterior resection | 0.907 | 0.956 | 0.965 |
| Positive circumferential resection margin | 0.650 | 0.475 | 0.505 |
| Prolonged length of stay | 0.804 | 0.767 | 0.897 |
| 2-year all-cause mortality | 0.093 | 0.075 | 0.022*Ϯ |
|  |  |  |  |
| **Surgeon-level** |  |  |  |
| 90-day mortality | 0.435 | 0.587 | 0.832 |
| 30-day unplanned re-admission | 0.324 | 0.289 | 0.402 |
| 30-day unplanned return theatre | 0.346 | 0.255 | 0.124 |
| Stoma at 18 months following anterior resection | 0.354 | 0.285 | 0.288 |
| Positive circumferential resection margin | 0.596 | 0.572 | 0.651 |
| Prolonged length of stay | 0.020* | 0.011* | 0.017* |
| 2-year all-cause mortality | 0.400 | 0.511 | 0.075 |
|  |  |  |  |

Ϯ The OR for the linear term was 1.02 (95% CI: 1.00 to 1.04) and for the quadratic term was 1.00 (95% CI: 1.00 to 1.00).
